# MLA Research Training Institute (RTI) 2018 and 2019: participant research confidence and program effectiveness

**DOI:** 10.5195/jmla.2024.1915

**Published:** 2024-10-01

**Authors:** Susan Lessick, Jodi L. Philbrick, Lorie Kloda

**Affiliations:** 1 slessick@uci.edu, Distinguished Librarian Emerita, UCI Libraries, University of California, Irvine, CA; 2 Jodi.Philbrick@unt.edu, Principal Lecturer, Department of Information Science, University of North Texas, Denton, TX; 3 lorie.kloda@mcgill.ca, Vice-Dean of Libraries. Libraries, McGill University, Montreal (Quebec), Canada

**Keywords:** Research confidence, research process, health sciences librarians, research training, librarian education, librarian research, evidence-based library and information practice (EBLIP)

## Abstract

**Objective:**

The article reports on an assessment of the effectiveness of the MLA Research Training Institute (RTI) for the years 2018 and 2019. The RTI is a year-long continuing education research methods training and support program for health sciences librarians. The study focuses on assessing RTI participants' research confidence after program completion and compares these results with their perceptions of workshop/program performance and learning outcomes. In addition, the authors discuss how the findings were applied to inform and improve the program.

**Methods:**

The study used a 26-item questionnaire, RTI Research Confidence Questionnaire, to gather information on participants’ self-reported research confidence before the workshop, immediately after the workshop, and one year after the workshop to determine statistically significant differences. Differences in research confidence were identified by using three nonparametric statistical tests. Additional workshop and program surveys were used to corroborate the research confidence findings.

**Results:**

Post-workshop and one-year-after-workshop research confidence ratings were significantly higher than pre-workshop levels for years 1 and 2. A comparison of median ratings between years 1 and 2 showed significant increases in research confidence for nine items in year 2. Participants’ positive perceptions of workshop/program effectiveness and learning outcomes corroborated these findings.

**Conclusion:**

Overall assessment findings indicated that RTI training helped participants understand, use, and apply research skills to conduct research. Findings also revealed that participants’ heightened research confidence persisted at least 12 months postintervention. The RTI Research Confidence Questionnaire proved effective for rigorously assessing and improving the RTI program. This study enhances the currently limited evidence on evidence-based approaches for assessing and improving research instruction for librarians.

## INTRODUCTION

Despite an increasing recognition of the importance of research by health sciences librarians, there have been few national initiatives to educate and support practicing health sciences librarians in conducting research. Moreover, there have been limited opportunities to address the wide range of issues facing health sciences librarians who are motivated to conduct research but are unfamiliar with and hesitant about the research process. As a way to redress these gaps, provide a comprehensive learning and support solution, and build the research capability of health sciences librarians on a national scale, the Medical Library Association (MLA) Research Training Institute for Health Sciences Librarians (RTI) was developed to help health sciences librarians build research skills, confidence, and experience to increase the quality, quantity, and dissemination of their research outputs.

The RTI builds on a distinctive and prescient vision from 30 years ago [1], which recognizes the importance of research in health information practice. This remains a foundational principle that continues to guide, challenge, and inspire contemporary health sciences librarians [2]. This influential MLA research policy statement [1], along with the second edition [3–4], that links research to health information practice, helped spark the rise of evidence-based library and information practice (EBLIP) [5], a conceptual framework that has continued to evolve and change with proponents of EBLIP introducing new EBLIP concepts, models, and dissemination platforms over time [2, 5–9]. Inspired by this heightened focus on advancing research in the profession and the subsequent discourse surrounding EBLIP, the research engagement and productivity of health sciences librarians increased over the span of several decades [5, 10–13].

Health sciences librarians benefit from using, creating, and applying research-based evidence in supporting quality health care and library practice. Health sciences librarians have played a key role in initiating, nurturing, and spreading evidence-based practice (EBP) in the health professions [5] and in fostering the growth of the EBLIP movement [5, 9, 14]. Librarian-led research has highlighted the positive contributions of health information provided by health sciences librarians and libraries on clinical decision-making and patient care [15–21], health professions' teaching and learning [17–18, 20–22], and systematic reviews [23–27]. Additionally, health sciences librarians have undertaken research to enhance library services and programs [13, 21, 28–29].

Despite these benefits, many librarians have struggled with conducting and publishing research studies. Journal editors and scholars have noted that the evidence base of health sciences librarianship needs strengthening in several areas, including an uneven quantity and quality of research across subject areas [4,10, 30–33]; an over-emphasis on quantitative and descriptive methods [30, 34–37]; and lower non-publication rates of conference abstracts [37–40]. Other studies have shown that health sciences librarians face various barriers and challenges to conducting and disseminating research, including a lack of research skills, training, and mentorship [4,13, 41–43] and a lack of research confidence [43–45], among other hindrances. More recently researchers have focused on research success factors to identify interventions for improving librarian research engagement. Several studies have found that research methods training [46–47], research confidence [48–49], and mentorship [50–54] have contributed positively to the research engagement and productivity of librarians.

Research-related education for health sciences librarians has long been identified as a necessary step for librarians to apply and engage in research. Early MLA research and education policies [1,55] emphasized the critical analysis and appraisal of published research findings to support healthcare users in clinical and academic settings. MLA's subsequent research policy [3] and professional competencies [56–57] shifted focus toward LIS research methods and cultivating a rich LIS research base. The promulgation of this influential research policy and MLA's professional competencies in research, coupled with the well-documented benefits of integrating research into practice, have led to an increased demand among health sciences librarian practitioners for continuing education options for research methods training with experienced researchers [13,58].

Several models of research training institutes for practicing librarians emerged in response to a demand for more in-depth research training. In 2012, the Canadian Association of Research Libraries began offering the Librarians' Research Institute (LRI) to practicing academic librarians in Canada [59]. In the U.S., two continuing education programs were established with grant funding from the Institute of Museum and Library Services (IMLS). The Institute of Research Design in Librarianship (IRDL), established in 2014, provides academic and research librarians with research methods training and support [60]. The Research Institute for Public Libraries (RIPL), launched in 2014, focuses on data and evaluation for public librarians [61].

The MLA Research Imperative Task Force (RITF), with the assistance of a planning group of research experts, developed a proposal to create the Research Training Institute for Health Sciences Librarians (RTI), which was approved by the MLA Board of Directors in 2016 [62]. Subsequently, MLA received federal funding from the Institute of Museum and Library Services (IMLS) Laura Bush 21^st^ Century Librarian Program to establish the MLA Research Training Institute for Health Sciences Librarians (RTI). The RTI is tailored to health sciences librarians' unique educational needs and focuses on advancing the creation, integration, and communication of health information research. In addition, it leverages MLA's educational, conference, and communication tools to maximize the participants' research learning environment, networking, and engagement.

This article examines the effectiveness of the RTI program during its initial two years, Year 1 (2018-2019) and Year 2 (2019-2020), with particular emphasis on changes in participants' research confidence and their perceptions of workshop/program performance and learning. Specifically, the article describes the results of assessing the effectiveness of the RTI summer workshop and continuing support in increasing and sustaining participants' research confidence after workshop completion and one year later. It compares and contrasts these results with survey data, evaluating participants' perceptions of workshop effectiveness and learning outcomes. The article also discusses how the findings were applied to inform and improve the RTI program. Additionally, it presents a brief literature review and description of the RTI program to provide context and rationale for the study.

### Description of the RTI Program

For the first two years, the RTI program focused on an in-person summer research methods workshop each July at the University of Illinois Chicago (UIC), followed by a year of formal mentoring and support as participants designed, conducted, and disseminated a research project. The weeklong workshop consisted of 12 learning modules with separate learning objectives, lectures, learning activities, small and large group discussions, and individual mentoring sessions. The RTI used MLA's MELIB-ED, an online education environment, to deliver content for all learning modules, including pre-recorded lectures, lecture slides, reading lists, schedules, syllabi, assignments, and worksheets.

The curriculum content for the workshop covered all the stages of the research process, including research planning and design, theoretical frameworks, method selection, data collection and analysis, dissemination of research, and reporting findings. Participants received expert instruction from five faculty instructors. All instructors were experienced librarian-researchers selected for their expertise and complementary research skills, as well as their experience teaching research to librarians and information studies students. The five faculty developed the initial curriculum content and delivered the online and in-person teaching activities.

Following the workshop and for the remainder of the institute year, participants participated in a mentoring program and two experiential learning activities (development of a research project and capstone presentation). Each participant was assigned to an experienced faculty/mentor who facilitated small group and individual mentoring sessions throughout the year. Each participant also actively participated in the RTI community of practice for their continued learning and collaboration, which provided online access to shared files, mentor forums, and forums for current and past cohort members. Participants completed a capstone experience at the end of each institute year by developing and presenting a poster on their research project at a special RTI session at the MLA annual meeting. Additionally, participants completed quarterly reports to monitor and support project completion.

The RTI's target audience was practicing librarians with novice and intermediate research skill levels who work in a wide variety of medical and non-medical work settings. RTI participants were selected for the program by a competitive process involving an independent double-blind review of applications by an MLA RTI jury appointed by the MLA Board of Directors, ensuring a fair and rigorous selection of applicants. Applicants were selected based on a publicly available rubric that evaluated the applicant's research learning goals, research proposal, professional achievements, and support letter from a supervisor. Seventy-three health sciences librarians from across the United States submitted applications for Years 1 and 2, and 20 RTI participants were selected to attend each of the two RTIs.

### Approaches To Assessing the RTI Program

Rigorous assessments took place throughout the program in both years: pre-workshop, during the workshop, post-workshop, and at quarterly intervals thereafter, including the end of the program. Areas assessed included participants' prior research engagement, research confidence, workshop/program performance, learning outcomes, program impacts, and research output. See Appendix A for an overview of all RTI areas assessed, methods used, and data collection time periods.

The RTI director and faculty used the assessment findings to evaluate the program and learning effectiveness, set goals for improvement for subsequent institutes, and help identify best practices in research learning and teaching. RTI data collection instruments and annual assessment findings are freely accessible online from the RTI website to promote transparency, allow for the verification and reuse of data, and ensure accountability to funding agencies and sponsors [63].

The focus of the present study assessed the program's effectiveness with special emphasis on changes in participants' research confidence and participant perceptions of workshop/program performance and learning. Specific questions that guided this study were:

1. Was the RTI effective in increasing the research confidence of participants post-workshop and one-year post-workshop?
2. Did the pre- and post-workshop research confidence of RTI participants increase in year 2 compared to year 1?
3. What were the perceptions of the RTI participants concerning key program performance and learning outcomes? Did these perceptions confirm or diverge from the research confidence findings?

## METHODS

This study used a quantitative approach to explore the effectiveness of the MLA RTI for years 1 and 2. Questionnaires were used to gather information on participants' self-reported research confidence. In addition, two quality improvement questionnaires were administered to gather information on the perceived performance of the RTI workshop/program, participants' perceptions of their learning, and to confirm results inferred from the research confidence data. The areas assessed in this study, including instruments used and timetable for each of these data collection points, are provided in Table 1.

**Table 1:** Areas Assessed, RTI Instruments Used, and Annual Timetable in Present Study

Areas Assessed	RTI Instrument Used	Annual Timetable
Research confidence (time 1)	RTI Research Confidence Questionnaire	Month before workshop (May)
Research confidence (time 2)	RTI Research Confidence Questionnaire	One month after workshop (August)
Workshop effectiveness	RTI Workshop Evaluation Survey	One month after workshop (August)
Research confidence (time 3)	RTI Research Confidence Questionnaire	One year after workshop (August following year)
Learning outcomes	RTI End-of-Program Evaluation Survey	One year after workshop (August following year)

RTI participants, who consisted of the study's population, were 40 health sciences librarians: 20 in year 1 and 20 in year 2. RTI participants came from 21 states across the United States and from a heterogeneous mix of libraries, including hospitals, academic health sciences, community colleges, health associations, and federal libraries. Participants were employed for an average of 10 years since completing their LIS master's degree and held a wide range of professional positions. 82% were female and 18% were male. All 40 RTI participants were invited to complete all assessments.

### Assessing Research Confidence

This assessment uses RTI participants' research confidence as an indicator of the effectiveness of the RTI research training and a predictor of their research success and productivity. Perceived research confidence, or research self-efficacy (RSE), is strongly associated with research training and mentoring in other fields [64]. Our research confidence assessment was informed by the work of Brancolini and Kennedy [65], who used Bandura's theory of self-efficacy [66] to develop, validate, and use a domain-specific research confidence instrument. The RTI Research Confidence Questionnaire (Appendix B) was adapted from their scale, the IRDL Librarian Research Confidence Scale (LRCS-38). The RTI Research Confidence Questionnaire includes a total of 26 items on research methods competencies, organized under the top-level phases of the research process.

The RTI Research Confidence Questionnaire was administered by an MLA staff member serving on the RTI Leadership Team via an online survey, Survey Monkey, a commercial online survey service. Blinded data was then sent to two authors for analysis. Differences in research confidence levels were identified by using three nonparametric statistical tests in SPSS statistical software (Version 28). The Wilcoxon Signed Ranks Test was used to determine if there were statistically significant differences in the self-reported research confidence of all participants before the workshop (time 1) and immediately after the workshop (time 2). The Friedman test assessed differences in the confidence levels of all participants across 3-time points: before the workshop (time 1), immediately after the workshop (time 2), and at the end of the one-year program (time 3) to assess the RTI's longer-term effectiveness and identify effective strategies that promote longer-term learning and retention. The Mann-Whitney *U* test was used to compare differences in research confidence between the RTI participants enrolled in year 1 to those in year 2 to determine if program adjustments made in year 2 based on multiple assessments improved the research confidence of participants. The statistical threshold was set at 0.05, p ≤ 0.05, for all tests.

### Assessing Workshop Effectiveness and Learning Outcomes

The RTI Workshop Evaluation Survey (Appendix C) consisted of 23, five-point Likert-scale items (1 - poor, 2 - below average, 3 - average, 4 - good, 5 - excellent) that asked participants to rate the effectiveness of specific aspects of the workshop. The RTI End-of-Program Evaluation Survey (Appendix D) consisted of 34 items and used a five-point Likert scale (1 - strongly disagree, 2 - disagree, 3 neither agree nor disagree, 4 - agree, 5 strongly agree) to measure participant perceptions of specific program elements and learning performance. This study uses four overall workshop effectiveness statements from the RTI Workshop Evaluation Survey (Q1, Q4, Q6-7) administered after the completion of the workshop and four learning performance statements from the RTI End-of-Program Survey (Q31-34) distributed at the end of the yearlong program. The four learning performance statements corresponded to four learning performance statements for funded IMLS Learning Award projects. Quality indicators for both surveys were a concurrence percentage of 80% or higher and a mean score of 4.0 or higher for all survey items. By using additional data sources from these two surveys, we compared and contrasted the workshop and learning results with the research confidence data to increase the reliability and robustness of our findings.

As data were collected with the limited intent of evaluating and improving the MLA RTI program, this study meets the definition of quality improvement and did not require approval by an institutional review board.

## RESULTS

### Research Confidence: Pre- and Post-Workshop (Wilcoxon Signed Ranks Test)

Analysis using the Wilcoxon Signed Ranks Test indicated that there were statistically significant differences in the research confidence ratings pre- and post-workshop for years 1 (N=20) and 2 (N =20), except for two items in year 2: Q7, “Assessing and synthesizing literature that is relevant to my research question;” and Q21, “Knowing how to manage the data.” See Table 2: Pre- and post-workshop research confidence for years 1 and 2 (Wilcoxon Signed Ranks Test).

**Table 2:** Pre- and post-workshop research confidence for years 1 and 2 (Wilcoxon Signed Ranks Test)

	Year 1				Year 2			
Questions about specific skills and knowledge needed for a research project.	*Mdn* (Pre, N=20)	*Mdn* (Post, N=20)	*Z* score	*p* value	*Mdn* (Pre, N=20)	*Mdn* (Post, N=20)	*Z* score	*p* value
1. Turning your topic into a question.	3	4	−3.087	.002*	3	4	−3.491	.000*
2. Designing a project to answer your question.	2.5	4	−3.630	.000*	3	4	−3.815	.000*
3. Selecting methods and procedures for your question.	2	3	−3.352	.001*	2	4	−3.971	.000*
4. Developing plan and timeline for your study.	2	4	−3.534	.000*	3	4	−1.973	.049*
5. Identifying appropriate information sources in which to conduct your literature search.	4	5	−2.221	.026*	2.5	5	−3.769	.000*
6. Using relevant keywords and search strategies to discover literature about the research topic.	4	5	−2.804	.005*	4	5	−2.299	.022*
7. Assessing and synthesizing literature that is relevant to your research question.	3	4	−2.984	.003*	4	4	−0.758	.448
8. Using a theoretical framework to inform the research design of your study.	1	3	−3.022	.003*	1.5	3	−2.702	.007*
9. Identifying sources of research funding and funding agency requirements.	2	3	−3.570	.000*				
10. Choosing an appropriate data gathering procedure.	2	3.5	−4.011	.000*	2	4	−3.787	.000*
11. Determining which members of a population to include in your study.	2	4	−3.672	.000*	2	4	−3.676	.000*
12. Knowing how to design a focus group.	2	3	−3.804	.000*	2	3.5	−3.903	.000*
13. Knowing how to run a focus group.	2	3	−3.682	.000*	2	3	−3.677	.000*
14. Knowing how to design an interview.	2	4	−3.685	.000*	2	4	−3.794	.000*
15. Knowing how to conduct an interview.	2	4	−3.499	.000*	2	4	−3.903	.000*
16. Knowing how to design a survey.	2	4	−3.839	.000*				
17. Knowing how to administer a survey.	2.5	4	−3.250	.001*	2.5	4	−3.703	.000*
18. Knowing institutional processes and standards to ensure that your study is conducted ethically.	3	4	−3.274	.001*	3	4	−3.469	.001*
19. Knowing what method of data analysis to use for your study.	1	3	−3.668	.000*	1.5	4	−3.872	.000*
20. Knowing what type of assistance you might need to undertake data analysis.	2	4	−3.809	.000*	1	4	−3.864	.000*
21. Knowing how to manage the data you have gathered.	2	3.5	−3.668	.000*	4	4	−.924	.356
22. Knowing how to code qualitative data to identify themes and subthemes.	1	3	−3.660	.000*	2	4	−3.560	.000*
23. Reporting results in written format.	2	3	−3.486	.000*	3	4	−3.787	.000*
24. Reporting results verbally.	2	3	−3.463	.001*	2.5	4.5	−3.677	.000*
25. Identifying appropriate places to disseminate results.	3	4	−3.640	.000*	3	4	−3.405	.001*
26. Tracking the dissemination and impact of your research.	3	4	−3.458	.001*	2.5	4	−3.072	.002*

* p value significant (p<0.05) by Wilcoxon Signed Ranks test.

Post-workshop research confidence ratings were **significantly higher** than pre-workshop research confidence for every item assessed for year 1. This result was the same for year 2, except for Q7 and Q21. The median ratings increased from 1.0 to 3.0 points, with the exception of Q7 and Q21 in year 2.

Pre-workshop median ratings for both years were relatively low for four items but were significantly higher post-workshop. Pre-workshop median ratings for both years were high for two items and remained high post-workshop. See Table 3: Pre-workshop research skills with low and high confidence ratings, years 1 and 2.

**Table 3:** Pre-workshop research skills with low & high confidence ratings, years 1 & 2

Q#	Skills with low confidence^*^	Q#	Skills with high confidence^†^
8	Using a theoretical framework	6	Using relevant keywords and search strategies to discover literature about the research topic
19	Knowing what method of data analysis to use for your study	7	Assessing and synthesizing literature that is relevant to your research question
20	Knowing what type of assistance, you might need to undertake data analysis		
22	Knowing how to code qualitative data to identify themes and subthemes		

* Research skills with pre-workshop ratings of ≤2 and post-workshop ratings of ≥*3*.

† Research skills with pre-workshop ratings of ≥*3* and post-workshop ratings of ≥4.

Post-workshop median ratings for both years were very high (medians of 5) for two items. Post-workshop increases of medians for both years were high (medians that rose 2+) for six items. See Appendix E: Table Post-workshop research skills with high medians and median increases, years 1 and 2.

### Research Confidence: Pre-, Post,- and One-Year-After-Workshop (Friedman Test)

Analysis using the Friedman Test indicated that there were statistically significant differences in the research confidence ratings pre-, post-, and one-year-after-workshop for years 1 and 2, with the exception of Q21 for year 2: Q21 *(Mdn 4*), “Knowing how to manage the data.” See Table 4: Pre-, Post-, One-year-after-workshop research confidence for years 1 and 2 (Friedman Test).

**Table 4:** Pre-, post-, and one-year-after-workshop research confidence for years 1 and 2 (Friedman Test)

	Year 1					Year 2				
Questions about specific skills and knowledge needed for a research project.	*Mdn* (Pre, N=20)	*Mdn* (Post, N=20)	*Mdn* (1 Yr, N=19)	χ^2^	*p* value	*Mdn* (Pre, N=20)	*Mdn* (Post, N=20)	*Mdn* (1 Yr, N=18)	χ^2^	*p* value
1. Turning your topic into a question.	3	4	4	14.392	.001^*^	3	4	4	15.125	.001^*^
2. Designing a project to answer your question.	2.5	4	4	28.836	.000^*^	3	4	4	26.793	.000^*^
3. Selecting methods and procedures for your question.	2	3	4	20.985	.000^*^	2	4	4	28.557	.000^*^
4. Developing plan and timeline for your study.	2	4	4	18.000	.000^*^	3	4	4	24.295	.000^*^
5. Identifying appropriate information sources in which to conduct your literature search.	4	5	5	7.190	.027^*^	2.5	5	5	14.659	.001^*^
6. Using relevant keywords and search strategies to discover literature about the research topic.	4	5	5	12.884	.002^*^	4	5	5	11.302	.004^*^
7. Assessing and synthesizing literature that is relevant to your research question.	3	4	5	14.982	.001^*^	4	4	4.5	12.667	.002^*^
8. Using a theoretical framework to inform the research design of your study.	1	3	3	25.581	.000^*^	1.5	3	3	14.880	.001^*^
9. Identifying sources of research funding and funding agency requirements.	2	3	3	19.500	.000^*^					
10. Choosing an appropriate data gathering procedure.	2	3.5	4	32.794	.000^*^	2	4	4	30.145	.000^*^
11. Determining which members of a population to include in your study.	2	4	4	22.164	.000^*^	2	4	4	20.698	.000^*^
12. Knowing how to design a focus group.	2	3	4	30.207	.000^*^	2	3.5	4	23.639	.000^*^
13. Knowing how to run a focus group.	2	3	4	30.145	.000^*^	2	3	4	20.109	.000^*^
14. Knowing how to design an interview.	2	4	4	28.444	.000^*^	2	4	4	26.655	.000^*^
15. Knowing how to conduct an interview.	2	4	4	25.240	.000^*^	2	4	4	27.898	.000^*^
16. Knowing how to design a survey.	2	4	4	27.263	.000^*^					
17. Knowing how to administer a survey.	2.5	4	4	19.478	.000^*^	2.5	4	4	26.308	.000^*^
18. Knowing institutional processes and standards to ensure that your study is conducted ethically.	3	4	5	20.491	.000^*^	3	4	5	22.291	.000^*^
19. Knowing what method of data analysis you would use for your study.	1	3	3	26.517	.000^*^	1.5	4	3	27.594	.000^*^
20. Knowing what type of assistance you might need to undertake data analysis.	2	4	3	25.200	.000^*^	1	4	4	25.733	.000^*^
21. Knowing how to manage the data you have gathered.	2	3.5	3	22.800	.000^*^	4	4	4	5.434	.066
22. Knowing how to code qualitative data to identify themes and subthemes.	1	3	3	26.281	.000^*^	2	4	4	26.333	.000^*^
23. Reporting results in written format.	2	3	4	20.481	.000^*^	3	4	4.5	23.705	.000^*^
24. Reporting results verbally.	2	3	4	21.893	.000^*^	2.5	4.5	4.5	23.186	.000^*^
25. Identifying appropriate places to disseminate results.	3	4	4	27.345	.000^*^	3	4	4.5	17.273	.000^*^
26. Tracking the dissemination and impact of your research.	3	4	4	19.538	.001^*^	2.5	4	4	19.283	.000^*^

* p value significant (p<0.05) by Friedman test.

One-year-after workshop research confidence ratings for year 1 (N=19) showed **significant gains** for every item over pre-workshop levels. This result was the same for year 2 (N=18), except Q21. Median ratings for both years increased by 0.5–3.0 points for every item one year after the workshop.

One-year-after-workshop median ratings for both years were high (medians with 5) for three items. One-year-after-workshop median increases for both years were high (medians that rose 2+) for ten items. See Table 5: One-year-after workshop research skills with high confidence ratings and median increases, years 1 and 2.

**Table 5:** One-year-after-workshop research skills with high confidence ratings and median increases, years 1 and 2

Q#	Research Skills
3	Selecting methods and procedures for your question^*^
5	Identifying appropriate information sources in which to conduct your literature search^†^
6	Using relevant keywords and search strategies to discover literature about the research topic^†^
10	Choosing an appropriate data gathering procedure^*^
11	Determining which members of a population to include in your study^*^
12	Knowing how to design a focus group^*^
13	Knowing how to run a focus group^*^
14	Knowing how to design an interview^*^
15	Knowing how to conduct an interview^*^
18	Knowing institutional processes and standards to ensure that your study is conducted ethically^*^^†^
22	Knowing how to code qualitative data to identify themes and sub-themes^*^
24	Reporting results verbally^*^

* Research skills with one-year-after-workshop median increases of ≥2.

† Research skills with one-year-after-workshop ratings of 5.

### Research Confidence: Year 1 Compared to Year 2 (Mann-Whitney U Test)

A comparison of median ratings between years 1 and 2 showed significant differences for nine items. Seven items showed statistically significant differences in year 2 at time 2 (post-workshop), and four items showed statistically significant differences in year 2 at time 3 (one year after the workshop). See Table 6: Research confidence comparison between years 1 and 2 (Mann-Whitney U).

**Table 6:** Research confidence comparison between years 1 and 2 (Mann-Whitney U Test)

Questions about specific skills and knowledge needed for a research project.	*U* (Pre) Time 1	*p* value	*U* (Post) Time 2	*p* value	*U* (1 Yr) Time 3	*p* value
1. Turning your topic into a question.	182.00	.596	170.00	.383	168.00	.918
2. Designing a project to answer your question.	192.00	.819	131.50	.062	162.00	.763
3. Selecting methods and procedures for your question.	173.00	.432	87.00	.001^*^	152.00	.541
4. Developing plan and timeline for your study.	164.50	.314	63.00	<.001^*^	159.00	.699
5. Identifying appropriate information sources in which to conduct your literature search.	194.00	.864	160.50	.203	162.00	.743
6. Using relevant keywords and search strategies to discover literature about the research topic.	174.50	.468	159.00	.334	144.00	.272
7. Assessing and synthesizing literature that is relevant to your research question.	188.00	.737	179.50	.549	162.00	.763
8. Using a theoretical framework to inform the research design of your study.	182.50	.603	167.50	.355	168.00	.921
9. Identifying sources of research funding and funding agency requirements.	198.50	.966	111.50	.011^*^	159.50	.714
10. Choosing an appropriate data gathering procedure.	170.00	.383	129.00	.038^*^	123.50	.120
11. Determining which members of a population to include in your study.						
12. Knowing how to design a focus group.	199.50	.988	197.00	.931	158.50	.684
13. Knowing how to run a focus group.	188.50	.738	187.00	.710	124.50	.137
14. Knowing how to design an interview.	188.00	.726	180.50	.772	148.00	.457
15. Knowing how to conduct an interview.	174.00	.449	192.50	.828	165.00	.846
16. Knowing how to design a survey.						
17. Knowing how to administer a survey.	186.00	.684	157.00	.177	139.50	.279
18. Knowing institutional processes and standards to ensure that your study is conducted ethically.	187.50	.723	170.00	.367	153.00	.528
19. Knowing what method of data analysis you would use for your study.	150.00	.113	143.00	.103	136.50	.390
20. Knowing what type of assistance you might need to undertake data analysis.	184.00	.638	178.50	.531	143.00	.371
21. Knowing how to manage the data you have gathered.	34.00	<.001^*^	194.00	.863	107.00	.042^†^
22. Knowing how to code qualitative data to identify themes and subthemes.	141.00	.074	169.50	.382	109.50	.049^†^
23. Reporting results in written format.	155.00	.204	94.00	.002^*^	90.00	.009^†^
24. Reporting results verbally.	160.50	.257	105.00	.007^*^	103.00	.028^†^
25. Identifying appropriate places to disseminate results.	176.50	.495	136.00	.048^*^	132.50	.205
26. Tracking the dissemination and impact of your research.	175.50	.497	198.00	.952	129.00	.266

p value significant (p<0.05) by Mann-Whitney U test.

* Seven research skills (Q3-4,9-10,23-25) showed statistically significant differences in year 2 at time 2 (post-workshop).

† Four skills (Q21-24) showed statistically significant differences in year 2 at time 3 (one year after workshop).

Participants in year 2 rated more items with high confidence (median scores of 4–5) post-workshop and one-year-after-workshop than those in year 1 (44 vs. 35). Additionally, participants in year 2 rated fewer items with low median ratings (median scores of 3–3.5) at these time points than participants in year 1 (6 vs. 17 items). See Appendix F: Table Median ratings comparison for years 1 and 2.

### Workshop Effectiveness and Learning Outcomes

Additional program outcome measures were examined related to participant perceptions of workshop effectiveness and learning outcomes. Participants in both year 1 (N=20) and year 2 (N=20) regarded four main areas of RTI workshop performance highly, encompassing their overall perceptions of the workshop, evaluation of RTI services and staff, assessment of the curriculum quality, and appraisal of the instructors' effectiveness. The ratings ranged from 95% to 100%, indicating that they perceived these aspects as excellent or good. See Appendix G Table Workshop effectiveness and learning outcome results for years 1 and 2.

Large majorities of participants in year 1 (N=19) and year 2 (N=18) strongly agreed or agreed with the four positive learning outcomes statements. Participants in both years strongly agreed or agreed that their interest in and understanding of research increased (ranging from 89%-100%) and that they were confident in applying what they learned and their ability to do research because of the RTI program (ranging from 82% to 95%). The four learning outcomes in both years had median scores of 5 (strongly agree), except one median rating of 4 in year 1. Quality indicators of workshop effectiveness and learning outcomes for both years exceeded our assessment targets of ≥80% and a median rating of ≥4. See Appendix G: Table Workshop effectiveness and learning outcome results for years 1 and 2.

## DISCUSSION

Post-workshop research confidence increased significantly compared to pre-workshop research confidence for all items assessed for year 1 and all items for year 2, except for two items that had high scores that remained stable. Likewise, research confidence increased significantly one year after the workshop over pre-workshop confidence levels for all 26 items for year 1 and 25 items for year 2, with 1 item in year 2 testing high and remaining stable for all three time points. Moreover, median increases of more than 2 occurred more frequently one year after the workshop (time 3) than immediately after the workshop (time 2), indicating that participants' research confidence was not only sustained but continued to increase in many instances one year later.

Overall assessment findings related to significant improvements in research confidence as a direct result of RTI participation indicated that the RTI training offered helped participants develop confidence in understanding, using, and applying research skills. A secondary outcome of this study was in participants' research confidence retention at least 12 months postintervention, measured using the same questionnaire 12 months after workshop completion. We confirmed the research confidence findings by using other data sources, which indicated that participants perceived aspects of the workshop as effective and were able to acquire knowledge to conduct research as a result of their RTI participation.

RTI post-assessment research confidence results resemble those reported for the IRDL research training program [65]. RTI participants showed significant or sustained increases in post-workshop research confidence compared to pre-workshop levels, including the 19 items that were comparable to IRDL items. A direct comparison of results with IRDL program evaluations was not possible since our study employed nonparametric statistical tests to assess differences in the research confidence of RTI participants across multiple time points and included additional questionnaire items that were specifically relevant to health sciences librarianship. We also diverged from the IRDL study in that we were interested in assessing the impact of training on longer-term research learning/confidence retention of librarian participants, i.e., the inclusion of a one-year-later period (time 3), which has not been studied previously. A search of the literature uncovered no other focused research self-efficacy assessments of librarian research methods and support programs other than the seminal research confidence work developed for the IRDL program [65]. Like the IRDL study, the RTI used results (evidence) from a research self-efficacy instrument developed for librarians, in our case, the RTI Research Confidence Questionnaire, to assess the effectiveness of the RTI research training program and to revise and improve RTI research instruction.

Using the RTI Research Confidence Questionnaire to assess participants' research confidence allowed us to take a detailed approach to evaluating and improving our research instruction. Through iterative questionnaires, we were able to identify areas needing improvement, implement adjustments, evaluate these revisions, and determine whether they improved post-workshop confidence from year 1 to year 2. Changes to the curriculum made between year 1 and year 2 based on multiple assessment measures included reorganization of some classroom content and bolstering learning activities on topics in which participants had lower confidence ratings. The RTI faculty paid particular attention to items that received the highest and lowest levels of confidence pre- and post-workshop (time 1 and time 2). The pre-workshop data for year 1 (time 1) indicated that participants felt highly confident about literature reviews. Based on this data, the faculty changed the lecture-based content on literature reviews to online pre-workshop content in year 2, creating more time during the in-person workshop for topics with lower confidence ratings. Confidence levels for literature reviews post-workshop (time 2) remained high in year 2. Two items with very low median ratings pre-workshop pertained to the topics of theoretical frameworks and data analysis; faculty augmented these lectures with additional learning exercises, small group discussions, and hands-on activities. Post-workshop ratings in year 2 suggest that these curricular additions had a positive effect on participants' confidence ratings in these areas, as they were higher than in year 1. Integrating statistical training into the curriculum proved challenging, and various strategies were used to enhance the curriculum in this area over time. Even though the confidence ratings for data analysis topics were among the lowest, items related to data analysis showed some of the greatest post-workshop increases.

A comparison of the results of the confidence ratings for years 1 and 2 revealed statistically significant increases in numerous areas between these two years. Changing select classroom content to online formats did not appear to lessen the research confidence of year 2 participants while adding select classroom content and learning activities in areas of lower confidence appears to have increased the research confidence of year 2 participants. Median overall ratings for year 2 participants were higher than for year 1 participants. Ratings of 3 (moderately confident) decreased from year 1 to year 2, and ratings of 4 and 5 (confident and very confident) increased from year 1 to year 2.

The significant and sustained gains in research confidence one year after the workshop were particularly noteworthy and encouraging. Several learning strategies were developed and refined over the two years to optimize participant retention of knowledge, skills, and confidence. These post-workshop learning supports included the ongoing availability of course content and lecture recordings for participants via MEDLIB-ED; increasing mentoring support via individual and small group sessions and listservs; developing a quarterly report form and process for participants to report and reflect on their research learning and progress and receive encouragement; and providing focused guidance and structure for participants completing their capstone project that consolidated and integrated participants' research learning with hands-on research experience at the end of each program year. Weaving these knowledge/confidence retention strategies into the fabric of the program provided participants with a richer engagement and more enduring grasp of research twelve months post-workshop completion.

## LIMITATIONS

Several study limitations merit discussion. Firstly, it is worth noting that this study evaluated a relatively small population (40 participants) of practicing health sciences librarians who were self-selected to apply to and were chosen based on selection criteria to participate in the RTI training program. The findings are not meant to be generalized to all health sciences librarians in the US who may be interested in research training. It does suggest, however, that the research confidence and output of motivated librarians can be positively impacted immediately, and after one year by participating in the RTI.

Secondly, due to the nature of self-report questionnaires, there is a possibility of response bias. Participants may not understand questions or misremember but answer anyway or may answer in a socially acceptable manner. The reinforcement of survey confidentially to encourage more accurate responses and the use of the same research confidence scale for generating data over different time periods provided greater confidence that our findings were valid and generalizable. We also used extensive follow-up procedures that limited the attrition bias of the one-year follow-up data for both years. In addition, the use of data triangulation allowed us to cross-validate findings with multiple data sources (research confidence data with workshop effectiveness and learning outcome data) and helped reduce the potential for bias, giving us further confidence in our study conclusions.

Thirdly, two random survey questions (Q9 and Q16) were inadvertently omitted in the post-assessment survey (time 2) for year 2. During the analysis, we did not draw comparisons, inferences, or conclusions based on this unintended missing data with other types of study data.

## CONCLUSIONS

The present study enhances the currently limited evidence on effective ways to train practicing health sciences librarians in planning, designing, and executing research. The authors could not find any studies that have examined research methods training models for health science librarians or the connection between research methods training and research confidence of health sciences librarians, and only two studies that applied research confidence concepts to academic librarian research training [65] and support services [67]. Our study amplifies the evidence-based approach and research confidence assessment work of the Institute for Research Design in Librarianship (IRDL) program. Like that program, the RTI Research Confidence Questionnaire proved effective for rigorously assessing and improving the RTI program. The RTI questionnaire was also useful for assessing the research confidence retention of RTI participants over time. The RTI faculty will continue to use and update the RTI Research Confidence Questionnaire and base programs on the results, thus using an evidence-based approach to evaluate and improve future research training programs for health sciences librarians. The RTI training, support, and assessment model can be informative for those designing, teaching, assessing, and improving research methods training in various disciplines, formats, and across educational levels. The model, which cultivates research education and self-efficacy, has proven to be an effective approach for building the research capacity of health information practitioners and serves as a promising foundation for advancing MLA's generational research vision.

## Data Availability

Data associated with this article are available in the Open Science Framework at https://doi.org/10.17605/OSF.IO/5MJYD.

## SUPPLEMENTAL FILES

**Appendix A:** Table Overview of RTI Areas Assessed

**Appendix B:** RTI Research Confidence Questionnaire

**Appendix C:** RTI Workshop Evaluation Survey

**Appendix D:** RTI End-of-Program Evaluation Survey

**Appendix E:** Table: Post Workshop Research Skills with High Medians Increases

**Appendix F:** Table: Median Rating Comparison

**Appendix G:** Table: Workshop Effectiveness Learning Outcoms
