# Supplementary material for: MLA Research Training Institute (RTI) 2018 and 2019: participant research confidence and program effectiveness
Source: J Med Libr Assoc. 2024 Oct 7;112(4):307–23. doi: 10.5195/jmla.2024.1915 (PMC11486066; doi:10.5195/jmla.2024.1915)
Supplement: Supplementary file 1 — Appendix A: Table Overview of RTI Areas Assessed [file jmla-112-4-307-s01.docx]

**Appendix A: Table Overview of RTI Areas Assessed, Methods, and Time Periods**

| Area assessed | Method | Time Periods |
| --- | --- | --- |
| Prior research engagement & research confidence (time 1) | Questionnaire | Before RTI begins |
| Workshop learning outcomes | Formative evaluations & feedback | During workshop |
| Workshop effectiveness | Questionnaire | Post-workshop |
| Research confidence (time 2) | Questionnaire | Post-workshop |
| Research confidence (time 3) | Questionnaire | One year post-workshop |
| Program effectiveness and learning outcomes | Questionnaire | One year post-workshop |
| Program impacts | Check-in messages and quarterly reports (self-report data) | Quarterly intervals |
| Research outputs | Individual communications with Director & reports (self-report data) | Ongoing |
