# Supplementary material for: MLA Research Training Institute (RTI) 2018 and 2019: participant research confidence and program effectiveness
Source: J Med Libr Assoc. 2024 Oct 7;112(4):307–23. doi: 10.5195/jmla.2024.1915 (PMC11486066; doi:10.5195/jmla.2024.1915)
Supplement: Supplementary file 2 — Appendix B: RTI Research Confidence Questionnaire [file jmla-112-4-307-s02.docx]

**Appendix B: RTI Research Confidence Questionnaire**

------------------------------------------------------------------------------------------------------------------

**On a scale of 1 to 5, with 1 being “Not at all confident” and 5 being “Very confident,” how would you rate your confidence in performing the following steps in a research project?**

**Scale: 1 = Not at all confident; 2 = Slightly confident; 3 = Moderately confident; 4 = Confident; 5 = Very confident**

**Planning Phase**

*1. Turning your topic into a question.

*2. Designing a project to answer your question.

*3. Selecting methods and procedures for your question.

*4. Developing a plan and timeline for the study.

*5. Identifying appropriate information sources in which to conduct your literature search.

*6. Using relevant keywords and search strategies to discover literature about the research topic.

7. Assessing and synthesizing literature that is relevant to your research question.

8. Using a theoretical framework to inform the research design of your study.

9. Identifying sources of research funding and funding agency requirements.

**Gathering Data Phase**

*10. Choosing an appropriate data gathering procedure.

*11. Determining which members of a population to include in your study.

*12. Knowing how to design a focus group.

*13. Knowing how to run a focus group.

14. Knowing how to design an interview.

15. Knowing how to conduct an interview.

*16. Knowing how to design a survey.

*17. Knowing how to administer a survey.

18. Knowing institutional processes and standards to ensure that your study is conducted ethically.

**Analyzing Data Phase**

*19. Knowing what method of data analysis to use for your study.

20. Knowing what type of assistance you might need to undertake data analysis (e.g., data/statistics consulting, transcription, software).

*21. Knowing how to manage the data you have gathered.

*22. Knowing how to code qualitative data to identify themes and sub-themes.

**Reporting and Impact Phase**

*23. Reporting results in written format.

*24. Reporting results verbally.

*25. Identifying appropriate places to disseminate results.

26. Tracking the dissemination and impact of your research.

*Questions that are the same or nearly the same as questions used in the Librarian Research Confidence Scale (LRCS-38) [65].
