# Supplementary material for: MLA Research Training Institute (RTI) 2018 and 2019: participant research confidence and program effectiveness
Source: J Med Libr Assoc. 2024 Oct 7;112(4):307–23. doi: 10.5195/jmla.2024.1915 (PMC11486066; doi:10.5195/jmla.2024.1915)
Supplement: Supplementary file 3 — Appendix C: RTI Workshop Evaluation Survey [file jmla-112-4-307-s03.docx]

**Appendix C: RTI Workshop Evaluation Survey**

**Please evaluate the RTI face-to-face workshop overall:**

**Rating scale: Excellent, Good, Average, Below Average, Poor, N/A.**

Q1 In-person workshop overall

Q2 Chicago, IL as a workshop destination

Q3 UIC library facilities

Q4 RTI/library services & staff

Q5 Pre-institute curriculum work

Q6 Overall curriculum quality (all modules)

Q7 Overall effectiveness of instructors

Q8 Lectures and discussions

Q9 Small group activities

Q10 Length of time for the workshop (overall)

Q11 Wi-Fi access at UIC

Q12 Social events

Q13 Breaks & refreshments

Q14 Comments or feedback about the in-person workshop?

**Please evaluate the RTI workshop resources:**

**Rating scale: Excellent, Good, Average, Below Average, Poor, N/A.**

Q15 MEDLIB-ED course materials

Q16 Printed materials (during in-person workshop)

Q17 RTI Community of Practice website on MLANET

Q18 RTI public website on MLANET

Q19 Pre-institute travel & logistics instructions/information

Q20 Comments or feedback about RTI workshop resources?

**Q21 Where did you stay during the Institute?**

- **UIC Single Student Residence (dormitory)**
- **Club Quarters Hotel**
- **Other (please specify)**

**Please evaluate your housing accommodations**

**Rating scale: Excellent, Good, Average, Below Average, Poor, N/A.**

Q22 Housing accommodations overall

Q23 Value for housing price paid

Q24 Proximity to UIC library

Q25 Proximity to dining and transportation options

Q26 Feeling of safety

Q27 Comments or feedback about housing?

**Q28 Were any relevant topics missing in your opinion? Please specify:**

**Q29 Please provide any additional suggestions to improve the Research Training Institute:**

**Q30 If you would like to provide a testimonial to be used in promoting an upcoming Research Training Institute, provide your comments below. If you would like RTI/MLA to acknowledge you as the author, please include your name (this area will not be used in data analysis). w**
