# Supplementary material for: MLA Research Training Institute (RTI) 2018 and 2019: participant research confidence and program effectiveness
Source: J Med Libr Assoc. 2024 Oct 7;112(4):307–23. doi: 10.5195/jmla.2024.1915 (PMC11486066; doi:10.5195/jmla.2024.1915)
Supplement: Supplementary file 4 — Appendix D: RTI End-of-Program Evaluation Survey [file jmla-112-4-307-s04.docx]

**Appendix D: RTI End-of-Program Evaluation Survey**

**Please indicate your level of agreement with the following statements about the RTI Community of Practice:**

| **Please indicate your level of agreement with the following statements about the RTI Community of Practice (CoP).** |
| --- |
| *Answer choices: Strongly agree; Agree; Neither agree nor disagree; Disagree; Strongly disagree* |
| Q1 The content of CoP posts was relevant. |
| Q2 The quantity of CoP posts was appropriate. |
| Q3 The CoP was easy to access and helped me stay connected to our group. |
| Q4 I was able to ask my colleagues for feedback on my research. |
| Q5 Overall, the CoP was helpful to my research progress. |
| Q6 The CoP made me feel like I belong to a research community. |
| Q7 Participating in the CoP makes me feel like I have a network of peers from other institutions with whom I can talk about research. |
| Comments (optional) What aspects of the CoP were most valuable? What aspects can be improved? |
|  |
| **Please indicate your level of agreement with the following statements about mentoring support after the workshop.** |
| *Answer choices: Strongly agree; Agree; Neither agree nor disagree; Disagree; Strongly disagree* |
| Q8 The number of contacts with my mentor was just about right. |
| Q9 My mentor was accessible. |
| Q10 My mentor provided constructive and useful feedback on my research. |
| Q11 My mentor was supportive and encouraging. |
| Q12 I was able to consult with my mentor to get help on a specific aspect of my research. |
| Q13 I was able to consult with my mentor to get guidance on publishing my research. |
| Q14 Overall, my mentor was helpful to my research progress. |
| Comments (optional) What aspects of mentoring were most valuable? What aspects can be improved? |
|  |
| **Please indicate your level of agreement with the following statements about quarterly reports and timelines for project goals.** |
| *Answer choices: Strongly agree; Agree; Neither agree nor disagree; Disagree; Strongly disagree* |
| Q15 The questions in the report were relevant and captured my research activities. |
| Q16 The report provided an opportunity to receive constructive feedback and encouragement. |
| Q17 The frequency of reports (e.g. quarterly reports) was just about right. |
| Q18 The timeline in the report for completing my research project was realistic. |
| Q19 Overall, completing the quarterly reports was helpful to my research progress. |
| Comments (optional) What aspects of the report were most valuable? What aspects can be improved? |
|  |
| **Please indicate your level of agreement with the following statements about your e-poster and presentation experience at the MLA meeting.** |
| *Answer choices: Strongly agree; Agree; Neither agree nor disagree; Disagree; Strongly disagree; N/A* |
| Q20 I received the information I needed to develop my eposter. |
| Q21 I received the information I needed to develop my presentation. |
| Q22 I had sufficient time to develop my eposter. |
| Q23 I had sufficient time to develop my presentation. |
| Q24 I enjoyed sharing my research knowledge and project with attendees during the RTI session. |
| Q25 Hearing the presentations of other Fellows during the RTI session helped me improve my presentation skills. |
| Q26 Hearing the research experiences and projects of other Fellows during the RTI session helped me enhance my research knowledge and skills. |
| Q27 I enjoyed interacting with Fellows, instructors, and attendees about research at the RTI session. |
| Q28 I enjoyed presenting my research project and interacting with the attendees in a virtual environment. |
| Q29 I am confident that I have the ability to design a research poster. |
| Q30 I am confident that I have the ability to give a research presentation. |
| Comments (optional) What aspects of the RTI session were most valuable? What aspects can be improved? |
|  |
| **Please indicate your level of agreement with the following statements about your learning experience as a result of MLA’s RTI program.** |
| *Answer choices: Strongly agree; Agree; Neither agree nor disagree; Disagree; Strongly disagree* |
| Q31 My understanding of research has increased as a result of the RTI program. |
| Q32 My interest in research has increased as a result of the RTI program. |
| Q33 I am confident I can apply what I learned in the RTI program. |
| Q34 I am confident that I have the ability to do research. |
| Comments (optional) What aspects of the RTI program were most valuable? What aspects can be improved? |
|  |
