# Supplementary material for: MLA Research Training Institute (RTI) 2018 and 2019: participant research confidence and program effectiveness
Source: J Med Libr Assoc. 2024 Oct 7;112(4):307–23. doi: 10.5195/jmla.2024.1915 (PMC11486066; doi:10.5195/jmla.2024.1915)
Supplement: Supplementary file 5 — Appendix E: Table: Post Workshop Research Skills with High Medians Increases [file jmla-112-4-307-s05.docx]

| **Appendix E: Table Post-workshop research skills with high medians and median increases, years 1 & 2** | |
| --- | --- |
| **Q#** | **Research Skills** |
| 5 | Identifying appropriate information sources in which to conduct your literature search* |
| 6 | Using relevant keywords and search strategies to discover literature about the research topic* |
| ** Research skills with pre-workshop ratings of 5*$.$ | |
| 11 | Determining which members of a population to include in your study† |
| 14 | Knowing how to design an interview† |
| 15 | Knowing how to conduct an interview† |
| 19 | Knowing what method of data analysis to use for your study† |
| 20 | Knowing what type of assistance, you might need to undertake data analysis† |
| 22 | Knowing how to code qualitative data to identify theme and subthemes† |
| *† Research skills with post-workshop median increases of* $\geq2.$ | |
