# Supplementary material for: MLA Research Training Institute (RTI) 2018 and 2019: participant research confidence and program effectiveness
Source: J Med Libr Assoc. 2024 Oct 7;112(4):307–23. doi: 10.5195/jmla.2024.1915 (PMC11486066; doi:10.5195/jmla.2024.1915)
Supplement: Supplementary file 6 — Appendix F: Table: Median Rating Comparison [file jmla-112-4-307-s06.docx]

**Appendix F: Table Median ratings comparison for years 1 and 2**

|  | **Year 1** | | | **Year 2** | | |
| --- | --- | --- | --- | --- | --- | --- |
| **Questions about specific skills and knowledge needed for a research project.** | ***Mdn***  **(Pre, N=20)** | ***Mdn***  **(Post, N=20)** | ***Mdn***  **(1 Yr, N=19)** | ***Mdn***  **(Pre, N=20)** | ***Mdn*  (Post, N=20)** | ***Mdn***  **(1 Yr, N=18)** |
| 1. Turning your topic into a question. | 3 | 4 | 4 | 3 | 4 | 4 |
| 1. Designing a project to answer your question. | 2.5 | 4 | 4 | 3 | 4 | 4 |
| 1. Selecting methods and procedures for your question. | 2 | 3 | 4 | 2 | 4 | 4 |
| 1. Developing plan and timeline for your study. | 2 | 4 | 4 | 3 | 4 | 4 |
| 1. Identifying appropriate information sources in which to conduct your literature search. | 4 | 5 | 5 | 2.5 | 5 | 5 |
| 1. Using relevant keywords and search strategies to discover literature about the research topic. | 4 | 5 | 5 | 4 | 5 | 5 |
| 1. Assessing and synthesizing literature that is relevant to your research question. | 3 | 4 | 5 | 4 | 4 | 4.5 |
| 1. Using a theoretical framework to inform the research design of your study. | 1 | 3 | 3 | 1.5 | 3 | 3 |
| 1. Identifying sources of research funding and funding agency requirements. | 2 | 3 | 3 | 2 |  | 3.5 |
| 1. Choosing an appropriate data gathering procedure. | 2 | 3.5 | 4 | 2 | 4 | 4 |
| 1. Determining which members of a population to include in your study. | 2 | 4 | 4 | 2 | 4 | 4 |
| 1. Knowing how to design a focus group. | 2 | 3 | 4 | 2 | 3.5 | 4 |
| 1. Knowing how to run a focus group. | 2 | 3 | 4 | 2 | 3 | 4 |
| 1. Knowing how to design an interview. | 2 | 4 | 4 | 2 | 4 | 4 |
| 1. Knowing how to conduct an interview. | 2 | 4 | 4 | 2 | 4 | 4 |
| 1. Knowing how to design a survey. | 2 | 4 | 4 | 2 |  | 4 |
| 1. Knowing how to administer a survey. | 2.5 | 4 | 4 | 2.5 | 4 | 4 |
| 1. Knowing institutional processes and standards to ensure that your study is conducted ethically. | 3 | 4 | 5 | 3 | 4 | 5 |
| 1. Knowing what method of data analysis you would use for your study. | 1 | 3 | 3 | 1.5 | 4 | 3 |
| 1. Knowing what type of assistance you might need to undertake data analysis. | 2 | 4 | 3 | 1 | 4 | 4 |
| 1. Knowing how to manage the data you have gathered. | 2 | 3.5 | 3 | 4 | 4 | 4 |
| 1. Knowing how to code qualitative data to identify themes and subthemes. | 1 | 3 | 3 | 2 | 4 | 4 |
| 1. Reporting results in written format. | 2 | 3 | 4 | 3 | 4 | 4.5 |
| 1. Reporting results verbally. | 2 | 3 | 4 | 2.5 | 4.5 | 4.5 |
| 1. Identifying appropriate places to disseminate results. | 3 | 4 | 4 | 3 | 4 | 4.5 |
| 1. Tracking the dissemination and impact of your research. | 3 | 4 | 4 | 2.5 | 4 | 4 |
|  |  |  |  |  |  |  |
| Total median scores | 59 | 96 | 102 | 64 | 96* | 106.5 |
| Number of items with median scores of 4-5 |  | 15 | 20 |  | 21 | 23 |
| Number of items with median scores of 3-3.5 |  | 11 | 6 |  | 3 | 3 |
| *No data for items Q#9 and Q#16. |  |  |  |  |  |  |
