# Supplementary material for: MLA Research Training Institute (RTI) 2018 and 2019: participant research confidence and program effectiveness
Source: J Med Libr Assoc. 2024 Oct 7;112(4):307–23. doi: 10.5195/jmla.2024.1915 (PMC11486066; doi:10.5195/jmla.2024.1915)
Supplement: Supplementary file 7 — Appendix G: Table: Workshop Effectiveness Learning Outcoms [file jmla-112-4-307-s07.docx]

**Appendix G: Table Workshop effectiveness and learning outcome results for years 1 and 2**

|  | **2018** | | **2019** | |
| --- | --- | --- | --- | --- |
| **RTI Participant Perceptions of Workshop Effectiveness** | **% Excellent & Good Rating (N=20)** | ***Mdn*** | **% Excellent & Good Rating (N=20)** | ***Mdn*** |
| Q1. Overall workshop | 95 | 5 | 100 | 5 |
| Q4. Overall RTI services and staff | 100 | 5 | 100 | 5 |
| Q6. Overall curriculum quality | 100 | 5 | 100 | 5 |
| Q7. Overall effectiveness of instructors | 100 | 5 | 100 | 5 |
| **RTI Participant Perceptions of Learning Outcomes** | **% Strongly Agree & Agree**  **(N=19)** | ***Mdn*** | **% Strongly Agree & Agree**  **(N=18)** | ***Mdn*** |
| Q31. My interest in research has increased as a result of the RTI program. | 95 | 5 | 89 | 5 |
| Q32. My understanding of research has increased as a result of the RTI program. | 95 | 5 | 100 | 5 |
| Q33. I am confident I can apply what I learned in the RTI program. | 95 | 5 | 94 | 5 |
| Q34. I am confident that I have the ability to do research. | 95 | 4 | 82 | 5 |
